# A Controlled Comparison of Human and AI-Assisted Automated Revision of Delphi Statements on RNA-Based Medicines: Parallel, 2-Arm Study

**DOI:** 10.2196/90228

**Published:** 2026-07-13

**Authors:** Enrico Nello, Fabio Tedone, Elena Caproni, Davide Cafiero, Sara Manellari, Paolo Rocco

**Affiliations:** 1 Helaglobe srl Firenze Italy; 2 Department of Pharmaceutical Sciences University of Milan Milano, Lombardy Italy

**Keywords:** Delphi method, large language models, retrieval augmented generation, RNA-based therapeutics, consensus, multi agent systems, artificial intelligence, AI

## Abstract

**Background:**

The Delphi method is widely used to derive expert consensus on complex clinical problems, yet it is slow and resource intensive. Recent advances in large language models and retrieval‑augmented generation (RAG) offer the possibility of accelerating consensus while maintaining methodological rigor. Large language models can retrieve and summarize evidence, but they frequently hallucinate and cannot reliably cite sources. At the same time, RNA‑based drugs and messenger RNA vaccines are rapidly moving from concept to clinic, generating a pressing need for timely, evidence‑based consensus on regulatory, manufacturing, and clinical issues.

**Objective:**

We evaluated whether a modular, RAG‑enabled, multi‑agent artificial intelligence (AI) pipeline could replicate the post–round 1 behavior of a human reviewer in a Delphi study. The primary objective was to determine whether AI‑assisted statement revision could rescue a greater proportion of subthreshold statements and achieve a consensus comparable to that obtained through human revision by round 2.

**Methods:**

A parallel, 2‑arm Delphi study was conducted on 28 statements about RNA medicines. In total, 50 international panelists (clinicians, researchers, and patient representatives) were randomized into human (arm A) and AI‑assisted (arm B) groups. After round 1, statements below the 75% agreement threshold were revised either manually by human reviewers or by an AI pipeline comprising the following software agents: (1) ReferenceDetector, to identify external citations; (2) Summarizer, to produce structured summaries of supporting PDFs; (3) a hybrid RAG module that combined dense and sparse retrieval with cross‑encoder reranking; and (4) Refiner, which generated revised statements, reasoning logs, and explicit citations. Two human reviewers with expertise in Delphi methodology and literature review who had contributed to statement development verified retrieved citations and approved or amended revisions. Agreement rates and vote distributions were compared across arms.

**Results:**

Arm A reached consensus on 71.4% (20/28) of the statements in round 1, whereas arm B reached consensus on 46.4% (13/28). After revision, consensus increased to 92.9% (26/28) of the statements in arm A and 85.7% (24/28) in arm B. The AI arm exhibited a larger mean improvement (absolute difference between rounds 1 and 2=39.3 percentage points) because more statements were initially below the threshold. Nonetheless, the absolute difference between arms after round 2 was modest (7.2 percentage points). AI‑assisted revisions were particularly effective for statements far below the threshold, but both arms failed to rescue 2 to 3 statements owing to substantive disagreements.

**Conclusions:**

A modular, citation‑anchored AI pipeline can closely approximate human performance in Delphi consensus procedures while substantially reducing manual workload. When paired with human oversight, AI assistance accelerated revision and closed most of the performance gap by the second round. Adoption of AI‑assisted workflows could accelerate consensus development on emerging technologies such as RNA therapeutics provided that transparency, rigorous retrieval, and human review are maintained.

## Introduction

The Delphi method is a structured technique for eliciting expert judgment and achieving consensus in complex domains [1]. Its strengths include anonymity of responses, controlled feedback, and the ability to draw on geographically dispersed experts [2]. These advantages make the Delphi method an attractive tool for health science questions for which empirical evidence is scarce. However, narrative reviews have emphasized persistent limitations: Delphi rounds are time‑consuming, often taking 2 to 6 months; panelist engagement declines with each round; recruiting and retaining qualified experts is difficult; and the heterogeneity of expert panels, while desirable for breadth of perspective, complicates data collection and consensus formation [2]. A recent scoping review of 287 Delphi studies found substantial heterogeneity in panel composition and modifications to the classic method, making comparisons across studies difficult and underscoring the need for methodological innovation [3].

In parallel, RNA-based therapeutics have moved rapidly from experimental platforms to clinical realities. Successes in developing messenger RNA vaccines against SARS‑CoV‑2 and respiratory syncytial virus, along with a pipeline of candidates for other infectious diseases, cancers, and rare diseases, have highlighted the flexibility of the platform but also exposed regulatory and societal challenges [4]. Regulatory thinking varies across jurisdictions, and public acceptance—the “social license to operate”—remains critical for uptake [4]. Timely, evidence‑based consensus statements can guide regulators, clinicians, and industry on the safe development and deployment of RNA-based products.

Large language models (LLMs) have been applied to medical knowledge retrieval and summarization. Retrieval‑augmented generation (RAG) integrates external knowledge into the generative process, enabling LLMs to answer domain‑specific questions. In a recent evaluation of 10 LLM‑RAG models for preoperative assessment, the GPT‑4 RAG model produced answers within 20 seconds and achieved 96.4% accuracy—significantly better than human reviewers (86.6%)—illustrating the potential of RAG to deliver rapid, accurate medical recommendations [5]. However, unaugmented LLMs often hallucinate, generating confident but fabricated statements and failing to provide verifiable citations [6]. These limitations have spurred interest in multi‑agent architectures that break down complex tasks into retrieval, synthesis, and verification phases, as well as hybrid retrieval systems combining dense and sparse methods. Hybrid RAG approaches have been shown to improve recall and ranking precision by leveraging both semantic and lexical signals [7,8].

Given the need for timely consensus on RNA therapeutics and the emerging capabilities of LLMs, we designed an artificial intelligence (AI)–assisted Delphi pipeline to mirror the workflow of a human reviewer in the post–round 1 phase. Our main objective was to compare consensus outcomes when subthreshold statements were revised manually vs through an AI pipeline that used RAG and multi‑agent modules. We hypothesized that AI assistance would rescue more statements by providing comprehensive evidence summaries and citation‑anchored revisions, whereas final consensus rates would approximate those of human reviewers.

Early explorations of AI‑facilitated Delphi exercises have highlighted both promise and pitfalls. Nóbrega et al [9] introduced AI Delphi, a machine-machine collaboration between multiple ChatGPT instances to explore future‑of‑work scenarios; the authors reported that LLMs could accelerate Delphi process execution but cautioned that human foresight remains indispensable. Perez Gálvez et al [10] subsequently proposed a hybrid soft consensus framework that treated generative LLMs as active participants alongside human experts; their experiments showed that LLM‑assisted panels can reach agreement faster but emphasized the need for careful governance and transparency. In a controlled chat‑based study, Triantafyllopoulos and Kalles [11] evaluated how LLMs facilitate agreement by adapting strategies such as clarification, summarization, and compromise; the authors found that GPT-4 produced consensus proposals more quickly than earlier models and argued for quantitative metrics to assess AI facilitation. Another Delphi‑style experiment used LLMs to forecast the evolution of generative AI and reported that AI‑mediated rounds captured diverse perspectives and mitigated respondent fatigue, although knowledge cutoffs and model biases remained challenges [12]. More recently, DeLLMphi has extended this line of work by constructing a fully in silico Delphi panel in which multiple “expert” LLMs, each conditioned on different superforecaster examples, iteratively update their forecasts in response to feedback from a mediator LLM. On a subset of the ForecastBench dataset, DeLLMphi outperformed alternative LLM configurations and closed approximately one-quarter of the performance gap between public forecasters and human superforecasters, highlighting the value of diversity, structured mediation, and multi-round interaction in LLM-based Delphi processes [13]. Together, these pilot studies suggest that generative models can enrich group decision‑making and speed consensus formation. However, these experiments are conducted in a relatively clean forecasting environment, where questions admit a numerical ground truth and where LLM agents never directly intervene in clinical or regulatory decisions. In contrast, Delphi exercises in health care often address normative statements under weak or fragmented evidence and rely heavily on tacit clinical experience. In such high-stakes settings, fully automated panels may be inappropriate; instead, AI agents are better framed as tools that retrieve and synthesize evidence while human experts retain responsibility for judgment and consensus formation.

## Methods

### Overview

This study used a parallel 2‑arm Delphi design. In total, 50 international panelists—including clinicians, researchers, and representatives of patient organizations—were recruited via professional networks, stratified, and randomized into 2 groups of 25 (50%) participants each to ensure a balanced distribution of professional backgrounds across the 2 arms (arm A: human revision; arm B: AI‑assisted revision). Before participation, panelists were informed that the study included both a human revision arm and an AI-assisted revision arm; however, they were not informed of their individual allocation and, therefore, did not know which revision process had been applied to the statements circulated within their arm. Panelists assessed 28 statements concerning the development, studies related to quality, and preclinical aspects and regulatory issues of the classification of RNA‑based medicines. Statements were rated on a 5‑point Likert scale (1=“strongly disagree”; 5=“strongly agree”). A consensus threshold was predefined as 75% or more of the panelists selecting “agree” or “strongly agree.” Round 1 remained open for 2 weeks. After closure, statements below the threshold entered the revision process. Operational end points such as turnaround time, reviewer workload, and cost were not prospectively measured as the study was designed to compare consensus outcomes after revision between arms. Panelists in both arms evaluated the same set of statements using the same 5-point Likert scale within the same 2-week response window; accordingly, panelist time commitment was intended to be comparable across arms.

**Figure 1:**
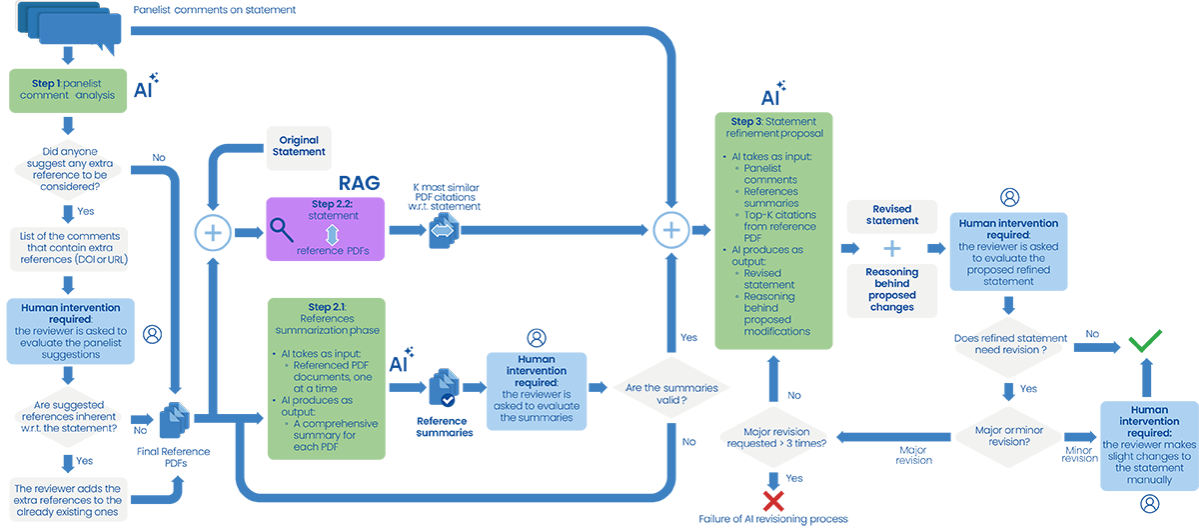
Modular artificial intelligence (AI)–assisted pipeline for revising subthreshold Delphi statements. Human reviewers verify external references and summaries, approve or amend AI‑generated revisions, and may request additional iterations. Major failures trigger the return to manual revision. DOI: digital object identifier; RAG: retrieval-augmented generation; w.r.t.: with respect to.

The AI pipeline (Figure 1) was designed to replicate five key steps undertaken by human reviewers: (1) analyzing panelist comments to detect requests for clarification or additional evidence, (2) identifying external references suggested by panelists, (3) summarizing supporting PDF documents, (4) retrieving text segments relevant to the statement, and (5) drafting a revised statement with a transparent rationale and explicit citations. Three specialized agents formed the core of the pipeline:

1. ReferenceDetector; this low‑temperature agent scanned comments for URLs, digital object identifiers, or explicit article titles and returned a list of candidate references along with a description of context. Detected references were presented to human reviewers for optional inclusion in the source library, ensuring that relevant evidence was considered without blindly trusting the model.
2. Summarizer; for each supporting PDF, a summarization agent generated a concise, structured synopsis at moderate temperature. Summaries were cached for reuse across statements to minimize redundant application programming interface calls and facilitate rapid comprehension. Human reviewers approved summaries before use.
3. Refiner; this agent received the original statement, percentage of agreement, panelist comments, retrieved evidence, and summaries. Operating at low temperature to prioritize accuracy, it drafted a revised statement, produced a change log explaining each modification, and generated a citation map linking edits to specific comments and evidence. The output adhered to a structured schema to facilitate human verification.

To ground revisions in verifiable evidence, the pipeline used a hybrid retrieval module. Text from the supporting PDFs was segmented and indexed using both dense embeddings (all‑MiniLM‑L6‑v2) and sparse BM25 representations.

The retrieval system operated on a static, curated source library composed of the supporting PDF documents available during the Delphi process together with any additional references suggested by panelists and validated by human reviewers before inclusion. The pipeline did not access regulatory databases or bibliographic sources in real time.

For each query (the subthreshold statement), both retrievers returned a fixed number of candidate passages. A cross‑encoder (ms‑marco‑MiniLM‑L‑6‑v2) reranked the combined candidates selecting the most relevant excerpts. During the hybrid fusion step, the final retrieval score was computed as a weighted combination of the individual retrievers, assigning a weight of 0.7 to the dense semantic scores and 0.3 to the sparse lexical (BM25) scores to favor semantic depth while retaining lexical coverage. Hybrid retrieval improves recall and precision compared with either dense or sparse retrieval alone; RAG combines pretrained sequence‑to‑sequence models with a dense vector index to generate more specific and factual answers [14]. Hybrid systems that fuse sparse BM25 and neural embeddings outperform either method individually in text retrieval tasks [7], and knowledge distillation from cross‑encoder rerankers yields dense representations that retain much of the accuracy of cross‑encoders while reducing storage requirements; when combined with sparse signals, these distilled embeddings approach cross‑encoder performance [8]. In biomedical search, hybrid first‑stage retrieval models combining lexical and neural methods consistently surpass purely sparse or purely neural models [15], and hybrid reranking schemes that aggregate similarity scores from dense and sparse retrievers deliver stable improvements over baseline rerankers [16]. We implemented our vector store using the Faiss library, which offers efficient algorithms for searching, clustering, and compressing vector representations [17]. Emerging work explores resource‑constrained hybrid retrieval architectures leveraging smaller language models to support RAG on edge devices, although these approaches were not tested in our pipeline [18].

At each stage, oversight was provided by 2 researchers with expertise in Delphi methodology whose familiarity with the topic derived from their involvement in the development of the statements through literature review. They were not panelists. In the AI-assisted arm, their role was limited to verification of retrieved references, summaries, and draft revisions rather than full manual interpretation of panelist comments and complete redrafting of statements. After reference detection and summarization, they confirmed that the references and summaries were appropriate. After retrieval and refinement, they examined the revised statement, change log, and citations. If a revision contained errors or omissions, they could request additional iterations or edit the statement manually. Reviewer time and cost were not prospectively recorded. This human‑in‑the‑loop design aligns with recommendations that LLM outputs be verified and that AI tools augment rather than replace expert judgment [19].

For each statement and round, the percentage of panelists selecting “agree” or “strongly agree” was calculated. Statements were classified as “passed” or “failed” based on the 75% or more threshold. We compared the number and proportion of statements passing in each arm across rounds using descriptive statistics. Because panelists evaluated the same statements across arms, no inferential tests were performed. Improvements were expressed as percentage‑point differences between rounds.

### Ethical Considerations

This methodological study involved an anonymous expert elicitation through a Delphi consensus procedure to evaluate aggregate statements on RNA-based medicines. Per the guidelines of local and institutional ethics committees regarding noninterventional research, formal ethics approval was not required as the study did not process individual patient health data, collect sensitive personal parameters, or involve clinical interventions on human participants. All participants were international health care experts, clinicians, and organizational representatives who took part voluntarily and were fully informed about the study’s scope, objectives, and data use protocols prior to completing the questionnaires. Completing the online feedback was considered informed consent to the use of aggregated, anonymized responses.

## Results

All 50 panelists completed the first round of voting. The human arm (arm A) reached consensus on 71.4% (20/28) of the statements, corresponding to items meeting or exceeding the 75% agreement threshold, whereas the AI‑assisted arm (arm B) reached consensus on 46.4% (13/28) of the statements. Mean Likert-scale scores were higher in the human arm (4.24, SD 0.82) than in the AI arm (3.90, SD 0.99), suggesting that participants in arm B were more critical at baseline. As a result, 28.6% (8/28) of the statements from arm A and 53.6% (15/28) of the statements from arm B required revision.

Human reviewers manually read comments, revisited references, and drafted edits. In the AI arm, the pipeline aggregated comments, detected missing references, retrieved relevant evidence, and produced citation‑anchored revisions with explicit rationales. Each AI-assisted revision was reviewed before circulation by 2 researchers; they verified retrieved citations and approved or amended the revised text.

An important aspect of the workflow was the “human‑in‑the‑loop” mechanism designed to call an expert reviewer whenever the pipeline suspected that commenters were requesting new references. Across all AI‑assisted revisions, the trigger was activated only once. In that instance, a panelist explicitly asked to include an additional reference, and the pipeline correctly flagged the comment. The human reviewer agreed and added the suggested reference, and the new citation was subsequently incorporated into both panels. There were no false-positive triggers (situations where the system called for human review even though no new references were requested) and no false negatives (instances in which a request for a new reference was missed). Once AI‑assisted revisions were approved, panelists rarely felt the need to intervene: none of the AI‑revised statements were rejected outright for being inaccurate or inconsistent with the literature, and only 4 comments required minor rewording. In 3 of these, the reviewer deleted a sentence beginning with “However,” deeming it unnecessary, and in 1 case, a brief parenthetical statement inserted by the AI was removed. Overall, the minimal editing required suggests that the AI pipeline produced revisions that were coherent, factually grounded, and aligned with panelist expectations.

**Figure 2:**
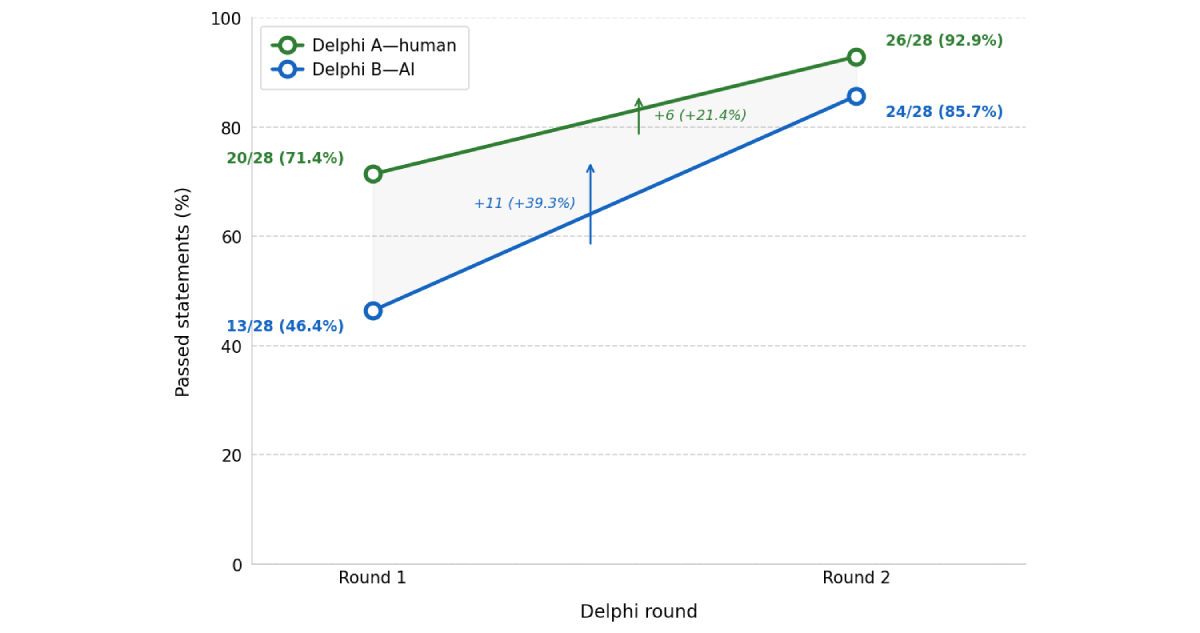
Cumulative pass rate per round. Labels indicate the number of statements passed and the percentage point increase from the previous round. The artificial intelligence (AI)–assisted arm exhibits a steeper slope owing to a lower starting point, but both arms converge by round 2.

After the revision phase, consensus improved markedly in both arms. Arm A went from 71.4% (20/28) of the statements passing in round 1 (95% CI 51.3%-86.8%) to 92.9% (26/28) in round 2 (95% CI 76.5%-99.1%), whereas arm B went from 46.4% (13/28; 95% CI 27.5%-66.1%) in round 1 to 85.7% (24/28; 95% CI 67.3%-96.0%) in round 2, closing most of the performance gap and leaving a modest absolute difference of 7.2 percentage points between the 2 arms after round 2. The AI-assisted arm exhibited a 39.3–percentage point increase in pass rate compared with a 21.4-point increase in the human arm, most likely because panelists in arm B were more critical at baseline, resulting in a lower round 1 pass rate and more statements entering revision below the threshold; residual heterogeneity in panel composition may also have contributed. Table 1 summarizes pass and fail counts before and after revision, and detailed statement-level results across both rounds are provided in Multimedia Appendix 1. Figure 2 illustrates the cumulative pass rate across rounds.

Throughout the experiment, the AI pipeline frequently delivered substantial improvements on statements that were far below the consensus threshold. For example, one statement regarding safety monitoring of RNA therapeutics, to which 16 of the 25 arm B panelists responded, increased from 43.8% (7/16) agreement to 75% (12/16) after AI revision, whereas the corresponding statement in the human arm showed little change. Another statement on regulatory harmonization improved by nearly 25 percentage points in the AI arm, reaching consensus alongside the human‑revised version. Nevertheless, not all statements benefited equally. A few complex items dealing with long‑term regulatory frameworks and public acceptance experienced minor declines after AI revision, underscoring the need for human judgment on nuanced topics. Overall, AI‑assisted revisions raised mean Likert-scale scores by roughly half a point.

Despite these gains, a small subset of statements remained below the 75% threshold after round 2—7.1% (2/28) in the human arm and 14.3% (4/28) in the AI arm. These outliers typically addressed contentious or speculative issues for which evidence was limited and on which panelists held divergent views. Panelist comments suggested that further rounds or alternative consensus methods would be required to resolve these disagreements.

**Table 1:** Pass and fail distribution per arm and round (n=28).

Arm	Round 1 passed, n (%)	Round 1 failed, n (%)	Round 2 passed, n (%)	Round 2 failed, n (%)
Human (arm A)	20 (71.4)	8 (28.6)	26 (92.9)	2 (7.1)
AI^a^ assisted (arm B)	13 (46.4)	15 (53.6)	24 (85.7)	4 (14.3)

^a^AI: artificial intelligence.

## Discussion

### Principal Findings

This controlled study demonstrates that a citation‑anchored, multi‑agent AI pipeline can nearly match human reviewers in rescuing subthreshold Delphi statements. The AI arm started from a lower baseline but achieved consensus on 85.7% (24/28) of the statements after 1 revision cycle, closing most of the gap with the human arm. AI‑assisted revisions were particularly effective for statements far below the threshold, suggesting that comprehensive retrieval and structured reasoning can surface evidence and clarify ambiguities that panelists may have overlooked. This is in line with recent AI-assisted post hoc assessment of published expert consensus showing that LLMs may help highlight convergent interpretations and identify statements deserving closer evidentiary review when evidence is limited while not replacing independent expert judgment [20]. Residual differences between arms were modest and may reflect panel heterogeneity rather than intrinsic limitations of the AI.

Our findings align with a growing body of evidence that RAG and hybrid retrieval substantially improve the reliability of LLM outputs. RAG couples a pretrained sequence‑to‑sequence model with a dense vector index so that the generation step can draw on external documents and, thus, produce more specific and factual answers [14]. Hybrid systems that fuse semantic (dense) and lexical (sparse) signals achieve superior recall and ranking precision compared with either method alone [7,8]. These results informed our design of a dense-sparse retrieval module and cross‑encoder reranking.

At the same time, unaugmented LLMs are prone to hallucinations. Adversarial evaluations of GPT‑4 and related models report hallucination rates ranging from 50% to 82%, and prompt engineering mitigations reduce hallucinations only modestly [19]. Such vulnerability underscores the need for retrieval mechanisms and human verification. Multi‑agent frameworks that break down tasks into retrieval, reasoning, and verification phases are increasingly advocated for to mitigate hallucinations and maintain factuality [6]. Our pipeline embodies this “retrieve-summarize-verify” paradigm: it anchors statements to cited evidence, exposes reasoning steps, and allows human reviewers to approve or amend each stage.

The inclusion of human reviewers is not merely precautionary; it reflects emerging guidance for transparent and accountable AI-assisted scientific writing and health-related AI use. In high-stakes consensus settings, AI can assist with retrieval, summarization, and draft revision, but decisions about the relevance of the retrieved evidence, its interpretation and grading, and the final normative framing of recommendations should remain under human responsibility. Reviewers, therefore, validate extracted references, approve summaries, and ensure that revised statements align with domain knowledge and the intended consensus context. This iterative oversight balances the efficiency gains of automation with the trustworthiness of expert judgment and is better understood as collaborative intelligence than as autonomous AI decision-making [19,21-23].

In the context of consensus methodology, our results corroborate the observation that panel composition profoundly influences consensus outcomes. Heterogeneous panels bring diverse perspectives but make consensus harder to achieve [2]. In our study, the AI arm panelists were more critical at baseline, as reflected by both a lower mean Likert-scale score and a lower proportion of statements meeting the consensus threshold in round 1. This likely resulted in more subthreshold statements entering the revision phase and, consequently, a larger apparent improvement after revision. A further probable explanation is residual heterogeneity in panel composition, which is known to influence Delphi outcomes by shaping how conservatively or permissively statements are judged. When these baseline differences are taken into account, the final consensus rates between arms were nearly identical. Our work, therefore, supports the feasibility of integrating AI tools into Delphi processes while underscoring the continued importance of careful panel selection and human expertise.

This study has several limitations. First, the AI pipeline used a specific LLM and retrieval configuration based on a static, curated knowledge base rather than real-time access to regulatory or bibliographic databases; performance may vary with different models, temperatures, retrieval strategies, or source update policies. While live access might improve the timeliness of revisions by incorporating newly published evidence or updated regulatory documents, the use of a static source library improved reproducibility, traceability, and consistency across statements in this controlled study. Second, operational efficiency was not formally quantified. We did not prospectively measure turnaround time, reviewer workload, personnel cost, or the time required from panelists in each arm. Although panelist participation was designed to be comparable across arms and the AI-assisted workflow shifted expert input from full manual rewriting toward verification and minor postediting, this study cannot make formal claims about time or cost savings. Future studies should prospectively compare time to revision, reviewer effort per statement, and cost-effectiveness between human-only and AI-assisted workflows. Third, the human and AI arms differed in baseline criticality, which partly explains the larger improvement in the AI arm. Fourth, our pipeline used a single model; multi‑model ensembles might further reduce variance but would increase computational costs.

Research should extend this evaluation across diverse domains, panel sizes, and consensus thresholds. Comparative studies exploring alternative retrieval strategies, prompt designs, agent configurations, and multi‑model ensembles could identify optimal architectures. Formal assessments of time savings, computational costs, and environmental impact will inform the practical viability of AI‑augmented consensus workflows. Developing automated metrics for citation fidelity and hallucination detection could enhance trust in AI outputs. Qualitative research into panelists’ perceptions of AI‑assisted revisions and their willingness to disclose AI involvement will help refine human‑in‑the‑loop designs and support responsible adoption. In the rapidly evolving field of messenger RNA therapeutics, AI‑assisted consensus methods may help regulators and clinicians keep pace with scientific advances.

### Conclusions

This study demonstrates that a modular, retrieval‑augmented AI pipeline can deliver revision outcomes comparable to those of human reviewers in a Delphi consensus exercise on RNA therapeutics. The pipeline rescued a greater proportion of subthreshold statements by aggregating panelist comments, retrieving relevant evidence, and producing citation‑anchored revisions. Human oversight remains essential: experts must verify references, validate summaries, and approve final wording. With appropriate safeguards, AI‑augmented workflows can accelerate consensus development in rapidly evolving fields.

## Appendix

Multimedia Appendix 1 Detailed statement-level results, panel participation, and agreement percentages across both Delphi rounds.

## Abbreviations

AI: artificial intelligence
LLM: large language model
RAG: retrieval-augmented generation
