# Supplementary material for: A Controlled Comparison of Human and AI-Assisted Automated Revision of Delphi Statements on RNA-Based Medicines: Parallel, 2-Arm Study
Source: JMIR Med Inform. 2026 Jul 13;14:e90228. doi: 10.2196/90228 (PMC13408467; doi:10.2196/90228)
Supplement: Multimedia Appendix 1 [file medinform_v14i1e90228_app1.docx]

- Delphi A: statements revisioning performed by humans
- Delphi B: statements revisioning performed by AI
- **Voting Scale**: 1-5

|  | **ROUND 1** | | | | | | **ROUND 2** | | | | | |  | |
| --- | --- | --- | --- | --- | --- | --- | --- | --- | --- | --- | --- | --- | --- | --- |
| **Stm N°** | **# Voters** | | **Agreement (%)** | | **Avg Vote** | | **# Voters** | | **Agreement (%)** | | **Avg Vote** | | **Δ Agreement (%)** | |
|  | **A** | **B** | **A** | **B** | **A** | **B** | **A** | **B** | **A** | **B** | **A** | **B** | **A** | **B** |
| **1** | 20 | 17 | 80.00% | 76.47% | 4.05 | 4.00 |  |  |  |  |  |  |  |  |
| **2** | 21 | 17 | 100% | 88.24% | 4.90 | 4.29 |  |  |  |  |  |  |  |  |
| **3** | 21 | 17 | 95.24% | 88.24% | 4.71 | 4.12 |  |  |  |  |  |  |  |  |
| **4** | 21 | 17 | 80.95% | 76.47% | 4.24 | 3.88 |  |  |  |  |  |  |  |  |
| **5** | 21 | 16 | 66.67% | 43.75% | 3.81 | 3.69 | 18 | 16 | 66.67% | 75.00% | 3.89 | 3.94 | 0.00% | +31.25% |
| **6** | 21 | 17 | 80.95% | 94.12% | 4.24 | 4.47 |  |  |  |  |  |  |  |  |
| **7** | 21 | 17 | 95.24% | 88.24% | 4.43 | 4.47 |  |  |  |  |  |  |  |  |
| **8** | 21 | 15 | 52.38% | 46.67% | 3.76 | 3.40 | 16 | 14 | 81.25% | 71.43% | 4.06 | 3.79 | +28.87% | +24.76% |
| **9** | 19 | 16 | 57.89% | 50.00% | 3.63 | 3.25 | 16 | 15 | 100% | 60.00% | 4.56 | 3.93 | +42.11% | +10.00% |
| **10** | 20 | 14 | 95.00% | 85.71% | 4.50 | 4.21 |  |  |  |  |  |  |  |  |
| **11** | 17 | 14 | 82.35% | 78.57% | 4.12 | 4.00 |  |  |  |  |  |  |  |  |
| **12** | 19 | 14 | 84.21% | 64.29% | 4.16 | 3.79 |  | 14 |  | 64.29% |  | 3.71 |  | 0.00% |
| **13** | 19 | 15 | 73.68% | 40.00% | 4.05 | 3.40 | 17 | 14 | 88.24% | 64.29% | 4.24 | 3.86 | +14.56% | +24.9% |
| **14** | 15 | 11 | 93.33% | 63.64% | 4.60 | 3.91 |  | 10 |  | 80.00% |  | 4.10 |  | +16.36% |
| **15** | 15 | 10 | 80.00% | 60.00% | 4.47 | 3.60 |  | 9 |  | 77.78% |  | 4.33 |  | +17.78% |
| **16** | 15 | 11 | 66.67% | 63.64% | 4.00 | 4.00 | 10 | 10 | 60.00% | 100% | 3.80 | 4.60 | -6.67% | +36.36% |
| **17** | 15 | 10 | 93.33% | 60.00% | 4.60 | 3.80 |  | 10 |  | 80.00% |  | 3.90 |  | +20.00% |
| **18** | 14 | 10 | 50.00% | 70.00% | 3.64 | 4.00 | 11 | 9 | 90.91% | 77.78% | 4.27 | 3.89 | +40.91% | +7.78% |
| **19** | 14 | 9 | 71.43% | 44.44% | 4.00 | 3.67 | 11 | 10 | 100% | 80.00% | 4.27 | 4.00 | +28.57% | +35.56% |
| **20** | 14 | 10 | 64.29% | 80.00% | 3.93 | 4.20 | 11 |  | 90.91% |  | 4.36 |  | +26.62% |  |
| **21** | 14 | 11 | 92.86% | 63.64% | 4.29 | 4.09 |  | 10 |  | 80.00% |  | 4.20 |  | +16.36% |
| **22** | 14 | 10 | 92.86% | 40.00% | 4.50 | 3.70 |  | 9 |  | 88.89% |  | 4.11 |  | +48.89% |
| **23** | 14 | 11 | 100% | 72.73% | 4.64 | 4.36 |  | 11 |  | 90.91% |  | 4.55 |  | +18.18% |
| **24** | 19 | 15 | 94.74% | 93.33% | 4.68 | 4.67 |  |  |  |  |  |  |  |  |
| **25** | 18 | 15 | 94.44% | 80.00% | 4.61 | 4.13 |  |  |  |  |  |  |  |  |
| **26** | 19 | 15 | 100% | 86.67% | 4.79 | 4.40 |  |  |  |  |  |  |  |  |
| **27** | 19 | 14 | 94.74% | 92.86% | 4.68 | 4.43 |  |  |  |  |  |  |  |  |
| **28** | 19 | 15 | 78.95% | 53.33% | 4.21 | 3.73 |  | 15 |  | 86.67% |  | 4.20 |  | +33.34% |
| **# stms passed** |  | | 20/28 | 13/28 |  | |  |  | 6/8  🡺 **26/28** | 11/15 🡺 **24/28** |  |  |  |  |
